# BelSmile: a biomedical semantic role labeling approach for extracting biological expression language from text

**DOI:** 10.1093/database/baw064

**Published:** 2016-05-12

**Authors:** Po-Ting Lai, Yu-Yan Lo, Ming-Siang Huang, Yu-Cheng Hsiao, Richard Tzong-Han Tsai

**Affiliations:** ^1^Department of Computer Science, National Tsing-Hua University, No. 101, Section 2, Kuang-Fu Road, Hsinchu, Taiwan 30013, Republic of China; ^2^Department of Computer Science and Information Engineering, National Central University, No. 300, Zhongda Road, Zhongli, Taoyuan, Taiwan 320, Republic of China and; ^3^Department of Clinical Laboratory Sciences and Medical Biotechnology, College of Medicine, National Taiwan University, No.1, Section 1, Renai Road, Taipei, Taiwan 10002, Republic of China

## Abstract

Biological expression language (BEL) is one of the most popular languages to represent the causal and correlative relationships among biological events. Automatically extracting and representing biomedical events using BEL can help biologists quickly survey and understand relevant literature. Recently, many researchers have shown interest in biomedical event extraction. However, the task is still a challenge for current systems because of the complexity of integrating different information extraction tasks such as named entity recognition (NER), named entity normalization (NEN) and relation extraction into a single system. In this study, we introduce our BelSmile system, which uses a semantic-role-labeling (SRL)-based approach to extract the NEs and events for BEL statements. BelSmile combines our previous NER, NEN and SRL systems. We evaluate BelSmile using the BioCreative V BEL task dataset. Our system achieved an F-score of 27.8%, ∼7% higher than the top BioCreative V system. The three main contributions of this study are (i) an effective pipeline approach to extract BEL statements, and (ii) a syntactic-based labeler to extract subject–verb–object tuples. We also implement a web-based version of BelSmile (iii) that is publicly available at iisrserv.csie.ncu.edu.tw/belsmile.

## Background

A biological network such as a protein–protein interaction network or a gene regulatory network is a unique way of representing a biological system. Investigation of such networks is an important task in the field of life science. However, the rapid growth of research publications makes it difficult to keep track of novel networks or update existing ones. Therefore, automatically extracting the biological events from literature and representing them with formal languages like Biological Expression Language (BEL; http://www.openbel.org/bel-expression-language)has become essential for studying biological networks.

**Figure 1.**
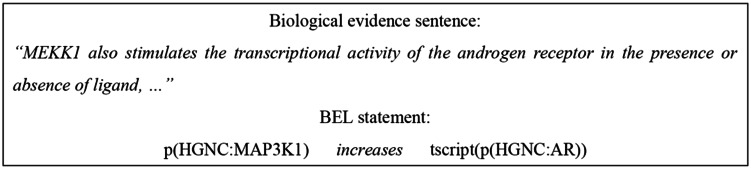
A BEL statement sample from the biocreative V BEL corpus.

BEL is one of the most popular languages for representing biological networks. It can indicate the causal and correlative relationships among biological entities (e.g. a chemical induces a disease). The entities’ identifiers, molecular activity and relation types can be described in a single statement that is easy for a trained life scientist to compose and understand. Figure 1 illustrates the BEL statement of the sentence ‘*MEKK1 also stimulates* *…*’*.* In the BEL statement, the protein is denoted by p() and the transcription activity is denoted by tscript(). The statement describes that the MEKK1 protein, whose HGNC symbol is MAP3K1, positively influences (‘increases’) the transcription of the androgen receptor, whose HGNC symbol is androgen receptor (AR). In a BEL statement, the named entity (NE) is also called an ‘abundance’, whereas the activity and relation type are called the ‘function’ and ‘predicate’, respectively.

In 2015, BEL was chosen by BioCreative V (1) as one of its information extraction tasks. The BioCreative V BEL task (1) includes two subtasks: (i) When a biological evidence sentence is provided, a text mining system should extract and return its BEL statement. (ii) When a BEL statement is provided, a text mining system should return a list of possible biological evidence sentences. In this study, we focus on the first subtask.

To automatically extract BEL statements with existing tools, the system needs to be capable of extracting different NE types such as proteins, chemicals, biological processes and diseases. It should also be able to normalize these NEs, classify them by their functions/activities and construct their causal and correlative relationships.

To quicken and facilitate the information gathering process for life scientists, this article describes the development of a semantic-role-labeling (SRL)-based system, BelSmile, for extracting BEL statements automatically. BelSmile is a pipeline approach that consists of four main tasks: (i) entity recognition, (ii) entity normalization, (iii) function classification and (iv) relation classification. As mentioned before, the offsets and boundaries of NEs are not available on the BEL corpora; therefore, BelSmile uses previous biomedical named entity recognition (NER) systems (2, 3) to recognize the NEs, and combines the results with dictionary-based recognizers. Subsequently, BelSmile uses a rule-based method to normalize NEs and classify their functions. Lastly, BelSmile uses a revised RCBiosmile (4), to extract subject–verb–object (SVO) tuples and determine the relation type. Each component of BelSmile will be explained in the following section.

## Materials and Methods

### System description

Our BelSmile system is a pipeline approach comprising four key stages: entity recognition, entity normalization, function classification and relation classification. First, we use our previous NER systems (2, 3, 5) to recognize the gene mentions, chemical mentions, diseases and biological processes in a given sentence. Second, the heuristic normalization rules are used to normalize the NEs to the database identifiers. Third, function patterns are used to determine the functions of the NEs. Finally, the SRL-based method classifies (4) the causal and correlative relationships.

### Entity recognition

BelSmile uses both CRF-based and dictionary-based NER components to automatically recognize NEs within the sentence. Each component is introduced as follows.

Gene mention recognition (GMR) component: BelSmile uses CRF-based NERBio (2) as its GMR component. NERBio is trained on the JNLPBA corpus (6), which uses the NE classes DNA, RNA, protein, Cell_Line and Cell_Type. Because the BioCreative V BEL task uses the ‘protein’ class for DNA, RNA and other proteins, we merge NERBio’s DNA, RNA and protein classes into a single protein class.

Chemical mention recognition component: We use Dai *et al.*’s approach (3) to recognize chemicals. Furthermore, we merge the BioCreative IV CHEMDNER training, development and test sets (3), remove sentences without chemical mentions, and then use the resulting set to train our recognizer.

Dictionary-based recognition components: To recognize the biological process terms and the disease terms, we develop dictionary-based recognizers that utilize the maximum matching algorithm. For recognizing biological process terms and disease terms, we use the dictionaries provided by the BEL task. In order to attain higher recall on protein and chemical mentions, we also apply the dictionary-based method to recognize both protein and chemical mentions. 

Table 1 summarizes the algorithm and resources used in different entity recognition components.

**Table 1.** The resources and models used for recognizing different entities

Type	Algorithm	ML Corpus	Dictionary
Biological process	Dictionary matching	—	BEL dictionary
Chemical	CRF and dictionary matching	BioCreative IV CHEMDNER	Chebi
Disease	Dictionary matching	—	BEL dictionary
Protein	CRF and dictionary matching	JNLPBA	Entrez gene

### Entity normalization

Following entity recognition, the NEs need to be normalized to their corresponding database identifiers or symbols. Given that the NEs may not exactly match their corresponding dictionary names, we apply heuristic normalization rules, such as converting to lowercase and removing symbols and the suffix ‘s’, to expand both entities and dictionary. Table 2 shows some normalization rules.

**Table 2.** Heuristic normalization rules

Rule	Examples
Basic rules	Converts to lowercaseRemoves hyphen, period, ahead ‘h’, ahead ‘human’ and ‘s’ behind the term
Parenthesis rules	Transforms ‘AAA(A)’ into ‘AAA—A’
Remove space rule	Transforms ‘IL 2 alpha’ into ‘IL2alpha’
Suitable rules	Removes general words such as ‘group’, ‘residue’, ‘protein’ and ‘atom’.
Stop word rules	Removes the preposition and article

Due to the size of the protein dictionary, which is the largest among all NE type dictionaries, the protein mentions are most ambiguous of all. A disambiguation process for protein mentions is employed as follows: If the protein mention exactly matches an identifier, the identifier will be assigned to the protein. If two or more matching identifiers are found, we use the Entrez homolog dictionary to normalize homolog identifiers to human identifiers.

### Function classification

In BEL statements, the molecular activity of the NEs, such as transcription and phosphorylation activities, should be determined by the BEL system. Function classification serves to classify the molecular activity.

We use a pattern-based method to classify the functions of the entities. A pattern can consist of either the NE types or the molecular activity keywords. Table 3 displays some examples of the patterns established by our domain experts for each function. If NEs are matched by the pattern, they will be transformed to their corresponding function statement.

**Table 3.** Examples of function patterns

Function	No. of Pattern	Pattern
molecularActivity (act)	15	<Protein> activity
complexAboundance (complex)	15	<Protein>/<Protein> complex
Degradation (deg)	11	<Protein> degradation
proteinModification (pmod)	9	phosphorylation of <Protein>
Translocation (tloc)	11	translocation of <Protein>

### SRL approach for relation classification

There are four types of relation in the BioCreative BEL task, including ‘increase’ and ‘decrease’. Relation classification determines the relation type of the entity pair. We use a pipeline method to determine the relation type. The method has three steps: (i) A semantic role labeler is used to parse the sentence into predicate argument structures (PASs), and we extract the SVO tuples from the PASs. (2) SVO and entities are transformed into the BEL relation. (3) The relation type is fine-tuned by the adjustment rules. Each step is illustrated below:

Step 1: Extracting SVO—Sentences are transformed into one or more PASs through SRL (described in the next section). Afterwards, the SVO is extracted from the PAS by mapping the predicate, agent and patient to the verb, subject and object, respectively.

Step 2: Extracting BEL statements—In the BEL task, the causal relationship is the ‘increase’/’decrease’ relation between two mentions, and it is similar to the regulation event types of BioNLP-ST (7–9). The regulation event keywords focus on types of gene regulation such as positive regulation and negative regulation, which are similar to the ‘increase’ and ‘decrease’ relations.

To classify the relation type, we select the regulation event terms from the BioNLP corpora (9), and our domain expert includes additional keywords for describing general causal relationships. Both event types ‘regulation’ and ‘positive_regulation’ are mapped to the relation type ‘increases’ in BEL, and the event type ‘negative_regulation’ is mapped to the relation type ‘decreases’ in BEL.

**Figure 2.**
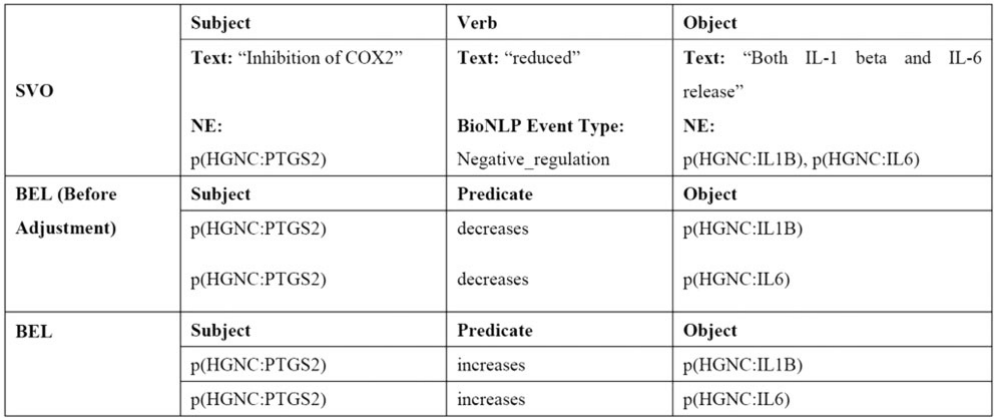
An example of transforming SVO into BEL statement.

As shown in Figure 2, entities which are inside the subject phrase or object phrase are mapped onto the subject or object in BEL, respectively.

Step 3: Adjusting BEL statements—in addition to being determined by the verb, relationship types are also determined by the words surrounding the NEs. Our domain expert collects a keyword list consisting of words that may alter the relationship type, such as ‘inhibition’, ‘mutant’ and ‘inactivation’. We use the keywords to adjust the relationship type accordingly. For instance, the relationship type in Figure 2 is ‘decreases’ before adjustment, while the context contains the keyword ‘inhibition’. The inhibition of p(HGNC:PTGS2) decreases both p(HGNC:IL1B) and p(HGNC:IL6), implying that p(HGNC:PTGS2) actually increases the level of both p(HGNC:IL1B) and p(HGNC:IL6). Therefore, the relationship type is changed from ‘decrease’ to ‘increase’.

### SRL component

**Figure 3.**
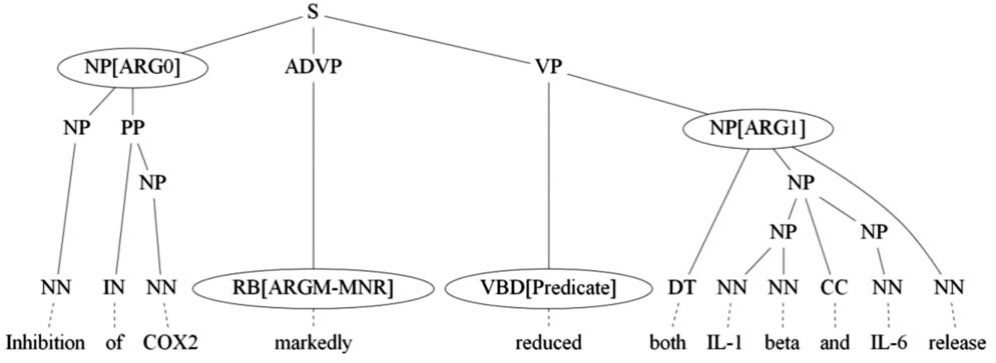
Example of a parse tree annotated with semantic roles.

Through SRL, the sentence can be represented by one or more PASs (10). Each PAS is composed of a predicate and several arguments. In our approach, the predicate is the verb, and the argument is a phrase of the sentence related to the predicate. The semantic role refers to the semantic relationship between a predicate and an argument of a sentence, which includes agent, patient, manner, location, etc. For example, the sentence in Figure 3, ‘Inhibition of COX2 markedly reduced both IL-1 beta and IL-6 release’, describes a molecular activation process. It can be represented by a PAS in which ‘reduced’ is the predicate, ‘Inhibition of COX2’ and ‘both IL-1 beta and IL-6 release’ comprise ARG0 (agent) and ARG1 (patient), respectively, with ‘markedly’ as the ARGM-MNR (manner). The SRL component used in our system consists of two components, RCBiosmile (4) and a syntactic-based labeler (SBL).

RCBiosmile is a Markov-Logic-Network (MLN)-based biomedical semantic role labeler that ‘employs’ patterns to select candidate semantic roles for each argument. It uses MLN (11) to learn and predict the semantic role of each argument. RCBiosmile is trained on BioProp (12), which only annotates the PASs of 30 selected biomedical predicates with the highest frequency. Hence, we developed a SBL for BelSmile to label the semantic roles of the rest of the verbs.

**Figure 4.**
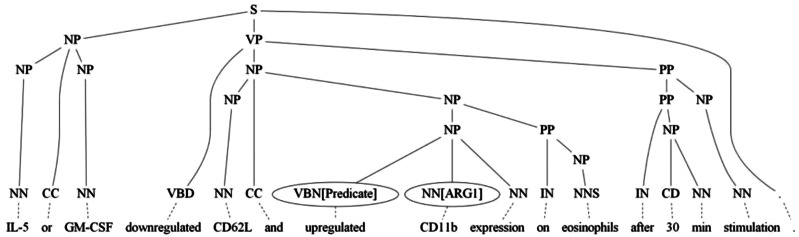
An example sentence with incorrect syntactic tree where two verbs ‘downregulated’ and ‘upregulated’ are in the sentence.

SBL: The SBL extracts the SVO from the sentence where the relation keywords were not covered by RCBiosmile. SBL uses a maximum-entropy (ME)-based SRL and a rule-based SRL. ME-based SRL formulates SRL as a constituent-by-constituent labeling task and uses the same feature set as BIOSMILE (13) except the features related to the predicate word. It is trained on BioProp. Additionally, a rule-based SRL is used to obtain the agent and patient, which might be missed by ME-based SBL. The rule-based SRL utilizes the syntactic tree to find the agent or patient of the verb. As shown in Figure 4, the agent which is ‘IL-5 or GM-CSF’ is missed by ME-based SRL. The rule-based SRL finds the verb’s grandparent S through the syntactic tree, and its left-side children contain NP, which is ‘IL-5 or GM-CSF’. As a result, the nearest child will be selected as the agent of the verb.

## Results

### Dataset

We use BioCreative V BEL corpus (14) to evaluate our approach. The corpus contains the BEL statements and the corresponding evidence sentences. The training set contains 6353 unique sentences and 11 066 statements, and the test set contains 105 unique sentences and 202 statements. One sentence may contain more than one BEL statement.

**Figure 5.**
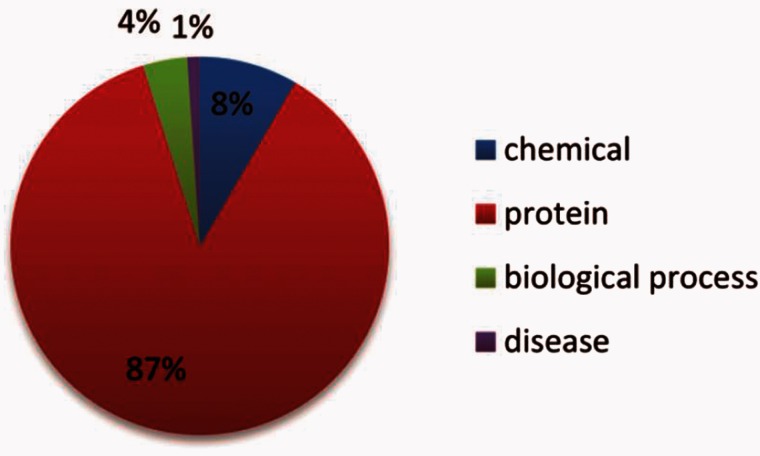
The distribution of the NE types in the training set.

**Figure 6.**
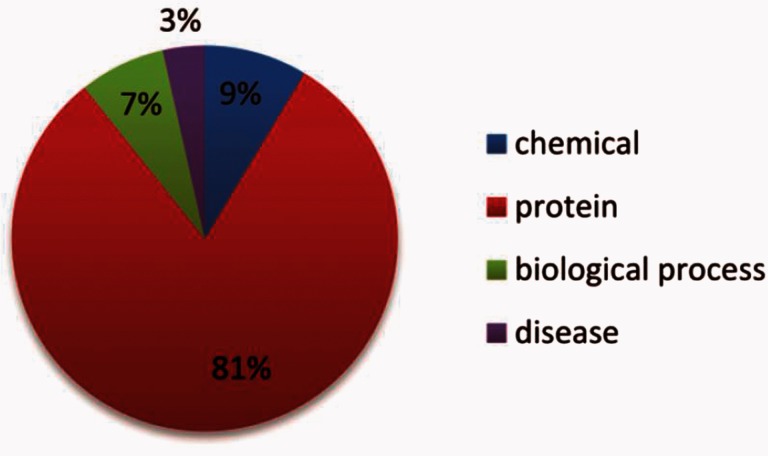
The distribution of the NE types in the test set.

NE types include: ‘abundance’, ‘proteinAbundance biologicalProcess’, pathology corresponding to chemical, protein, biological process and disease, respectively. Their distributions within the datasets are shown in Figures 5 and 6.

### Evaluation metrics

The F1 measure is used to evaluate the BEL statements (15). For term-level evaluation, only the correctness of NEs is evaluated. NEs are regarded as correct if the identifiers are correct. For function-level evaluation, the correctness of the discovered function is evaluated. Functions are correct when both the NE’s identifier and function are correct. As for the relationship-level evaluation, only the NEs and the relationships are considered. Relation is correct when both the NEs’ identifiers and the relationship type are correct. For the BEL-level evaluation, the NEs’ identifiers, function and the relationship type are all required to be correct for a true positive case.

## Result

The performance of each level is shown in Table 4, including the performance with gold NEs. The detailed performances for each type are shown in Table 5, and we evaluate the performances of RCBiosmile, ME-based SRL and rule-based SRL by removing them individually, and the relation-level result is shown in Table 6.

**Table 4.** The overall performance of each level

Evaluation metric	Precision	Recall	F-score
Term-level	68.45	34.11	45.53
Term-level with gold terms	100	35.78	52.70
Function-level	55.55	7.57	13.33
Function-level with gold terms	90.00	13.63	23.68
Relation-level	43.00	21.50	28.67
Relation-level with gold terms	70.67	26.50	38.55
BEL-level	42.00	20.79	27.81
BEL-level with gold terms	69.33	25.74	**37.55**

**Table 5.** The performances of each type

	Test set			Test set with gold NE		

**Entity type**	**P(%)**	**R(%)**	**F(%)**	**P(%)**	**R(%)**	**F(%)**
Biological process	100	8.7	16.0	100	8.7	16.0
Chemical	54.5	50.0	52.1	100	50.0	66.7
Disease	57.1	33.3	42.1	100	16.7	28.6
Protein	71.1	35.0	46.9	100	37.9	55.0

**Function type**	**P(%)**	**R(%)**	**F(%)**	**P(%)**	**R(%)**	**F(%)**

molecularActivity	25.0	3.2	5.7	66.7	6.5	11.8
complexAboundance	0	0	0	100	8.3	15.4
degradation	100	40.0	57.1	100	40.0	57.1
proteinModification	100	16.7	28.6	100	16.7	28.6
translocation	100	14.3	25.0	100	14.3	25.0

**Relation type**	**P(%)**	**R(%)**	**F(%)**	**P(%)**	**R(%)**	**F(%)**

increases	42.9	21.3	28.4	66.1	23.8	35.8
decreases	43.5	22.2	29.4	84.2	35.6	50.0

**BEL type**	**P(%)**	**R(%)**	**F(%)**	**P(%)**	**R(%)**	**F(%)**

increases	41.6	20.4	27.4	64.3	22.9	33.8
decreases	43.5	22.2	29.4	84.2	35.6	50.0

**Table 6.** The relation-level performances of removing individual SRL


	Test set with gold NE			
Relation type	P(%)		R(%)	F(%)
BelSmile	70.67		26.5	38.55
BelSmile remove RCBiosmile	70.27		26.0	37.96
BelSmile remove ME-based SRL	72.22		26.0	38.24
BelSmile remove Rule-based SRL	73.24		26.0	38.37

We retrieved the boundaries of abundances and processes by mapping the identifiers to the sentences with their synonyms in the database. As for gene names, if it cannot be mapped to the sentence, we map it to the NE with the smallest distance between two Entrez IDs, as they possess similar morphology. For instance, the Entrez ID of ‘heat shock protein family A (Hsp70) member 4’ is 3308, and that of ‘heat shock protein family A (Hsp70) member 5’ is 3309, while both IDs refer to the gene name ‘Hsp70’.

For term-level evaluation, we achieved an F-score of 45.53%. Since BelSmile focuses on extracting BEL statements in the SVO format, if the NEs recognized by our NER and normalization components are not within the subject or object, then they will not be output, resulting in a lower recall. Error cases due to the non-SVO format will be further examined in the discussion section. Moreover, the BEL dataset only contains mentions which are in the BEL statements, so those which are not in the BEL statements become false positives. For example, the ground truth of the sentence ‘L-plastin gene expression was positively regulated by testosterone in AR-positive prostate and breast cancer cells’. is ‘a(CHEBI:testosterone) increases act(p(HGNC:AR))’. Because the ‘p(HGNC:LCP1)’ recognized by BelSmile is not in the ground truth, it becomes a false positive.

For function-level evaluation, our approach achieved a relatively low F-score of 13.33%, owing to the fact that some function statements have no function keywords. For instance, the sentence ‘Glyceraldehyde-3-phosphate dehydrogenase (GAPDH) and triosephosphateisomerase (TPI) are essential to glycolysis’ has the ground truth of ‘act(p(HGNC:GAPDH)) increases bp(GOBP:glycolysis)’ and ‘act(p(HGNC:TPI1)) increases bp(GOBP:glycolysis)’. However, there is no function keyword of act (molecularActivity) for both ‘act(p(HGNC:GAPDH))’ and ‘act(p(HGNC:TPI1))’ in the sentence. As for the relation-level and BEL-level evaluation, we achieved F-scores of 28.67% and 27.81%, respectively.

### Comparison with other systems

Choi *et al.* (16) used the Turku event extraction system 2.1 (TEES) (17) and co-reference resolution to extract BEL statements. They achieved an F-score of 20.2%. Liu *et al.* (18) employed the PubTator (19) NE recognizer and a rule-based approach to extract BEL statements and achieved an F-score of 18.2%. Their systems’ performance along with the statement-level performance of BelSmile are presented in Table 7. BelSmile attained a recall/precision/F-score (RPF) of 20.3%/44.1%/27.8% in the test set, outperforming both systems. In the test set with gold NEs, Choi *et al.* (1) achieved an F-score of 35.2%, Liu *et al*. (2) attained an F-score of 25.6%, and BelSmile reached an F-score of 37.6%.

**Table 7.** The statement-level performances on BEL test set

	Test set			Test set with gold NEs		
	R(%)	P(%)	F(%)	R(%)	P(%)	F(%)
Choi *et al*.	12.4	54.4	20.2	23.8	67.6	35.2
Liu *et al*.	13.9	26.4	18.2	21.3	32.1	25.6
BelSmile	20.79	42.0	27.81	25.74	69.33	37.55

## Discussion

### Low performances on function-level evaluation

In Table 4, the F-score of BEL-level is 27.81%, however, the F-score of function-level is only 13.33%. According to our analysis on test set, there are 66% of sentences do not contain functions in the test set. In these sentences, our BEL-level performance is 37.5%. However, our BEL-level performance is lower than 5.1% in the other 34%. Therefore, the performance of the function-level is lower than that of the BEL-level. In Table 5, scores of molecularActivity and complex are both very poor. The reason is illustrated as follows. molecularActivity consists of several sub-types including catalyticActivity, kinaseActivity, transcriptionalActivity and transportActivity. Since our patterns were designed for the general molecularActivity category, not for each subcategory, 50% functions are predicted as molecularActivity, making the performance on this category molecularActivity the poorest. Most extracted functions are false positives. After removing these FPs by checking the gold-standard protein mentions, the precision is improved significantly.

### Error of temporal relation statement

A common error in temporal sentences is shown in the following two examples:

‘Following i.v. infusion of LPS into mice, up-regulation of C5aR occurred in the capillary endothelium of mouse lung’.

‘Finally, the abundance of MBD3 was highest in the late S phase when the DNMT1 is also most abundant, whereas the MBD2 level was largely constant throughout the cell cycle’.

In these two sentences, ‘Following i.v. infusion of LPS into mice’ and ‘when the DNMT1 is also most abundant’ are temporal arguments. The first implies that ‘LPS’, a(CHEBI:lipopolysaccharide), increases ‘C5aR’, p(HGNC:C5AR1). The second implies that ‘cell cycle’, bp(GOBP: ‘cell cycle’), increases ‘MBD3’, p(HGNC:MBD3). However, the system fails to detect the subject or object in the temporal argument, resulting in two false negatives. According to our observation on test set, ∼7.9% BEL statements are temporal relations.

### Error of location relation statement

Another error type is related to the location, as shown in this sentence:

‘We demonstrated the enhanced glycerol kinase enzymatic activity in Aqp7-KO and -knockdown adipocytes’.

In this example, ‘in Aqp7-KO and -knockdown adipocytes’ is the location argument. It implies that ‘Aqp7’, p(HGNC:AQP7), decreases ‘glycerol kinase enzymatic activity’, act(p(HGNC:GK)). However, the subject or object which is in the location argument is not detected, resulting in a false negative. According to our observation on test set, ∼7.4% are such statements.

### Related work

In this section, we give a brief review of core natural language processing components that are important in the BEL extraction task.

### Biomedical semantic role labeling

Biomedical semantic role labeling (BioSRL) is a natural language processing technique that identifies the semantic roles of the words or phrases in sentences describing biological processes and expresses them as PAS’s.

BioSRL is usually formulated as a supervised machine learning problem that relies on manually annotated training corpora (4, 13). However, building such large corpora requires much human effort. BioKIT (20) is a SRL system uses a SRL model trained using domain adaptation techniques and data from the Propbank (21) and Bioprop corpus (22).

Both PropBank and BioProp only annotate the verbal predicates, and both of them annotate arguments on the nodes of syntactic trees. Bethard *et al*. (23) proposed a BioSRL approach for protein transport that identifies both verbal and nominal predicates. They formulate BioSRL as a phrase-by-phrase labeling problem and use a word-chunking package, YamCha (24), to train their model.

### BioNLP shared task

Recently, several biomedical event extraction tasks (7, 8) have been proposed, and the BioNLP-ST 2013 Pathway Curation task (9) is one of the most important tasks among them. It is organized by University of Manchester’s National Centre for Text Mining (NaCTeM) and the Korea Institute of Science and Technology Information (KISTI). There are two aims of this task. The first is to evaluate performance of biological event extraction systems in supporting the curation, evaluation and maintenance of bio-molecular pathway information. The second is to encourage further improvement of biological event extraction methods and technologies. The 2013 Pathway Curation task provides a benchmark dataset where pathway-related entities—such as chemical mentions, gene mentions, complex and cellular components, and biological events (e.g. regulation and phosphorylation)—are also annotated in the training set and development set.

The participants can develop their event extraction systems on the dataset. A representative example is the Turku event extraction system 2.1 (TEES) (25), an event extraction system developed on BioNLP-ST 2013 corpus (8). To extract events, it uses McCCJ (26) to parse the sentences and convert them into the collapsed CCprocessed Stanford dependency scheme (27). It classifies event type with support vector machines using features generated from dependency tree information.

BioNLP-ST is similar to the BioCreative BEL task (1). Both of them focus on extracting the relationships between proteins, chemicals, diseases and other biomedical entities, and also on biomedical events such as phosphorylation and transcription. The main difference is that the BioNLP task only focuses on relation extraction; therefore, the offsets and NE types are given in the training, development and test sets. By contrast, the BEL task focuses not only on relation extraction but also on NER and normalization. Thus, BEL data do not include NE offsets, types, and identifiers, and systems need to be capable of integrating different BioNLP components such as the GMR/normalization and relation extraction tools.

## Conclusion

This article describes the construction of BelSmile, a system that can automatically extract BEL statements. BelSmile incorporates several previous BioNLP systems including NERBio, Dai *et al*.’s chemical name recognizer and RCBiosmile. Due to the limitation of the BioProp corpus, RCBiosmile only considers the 30 predicates with the highest frequency. In light of this, our SBL can label the subject-verb-object for predicates that are not covered by RCBiosmile. Furthermore, we also performed a thorough error analysis of SRL-based BEL statement extraction, hoping to assist those who are interested in the BEL task. We evaluated our approach on the BioCreative V BEL corpus. It achieved an RPF of 24.8/78.1/37.6 on the test set, and outperformed the BioCreative V BEL benchmark systems.
